# Severe outcomes and risk factors of non-neutropenic fever episodes in hospitalized children with cancer in Kenya

**DOI:** 10.3389/fonc.2025.1575714

**Published:** 2025-05-12

**Authors:** Samuel Kipchumba, Kenneth Busby, Dennis Njenga, Julia Dettinger, Lenah Nyamusi, Sandra Langat, Gilbert Olbara, Cheryl A. Moyer, Terry A. Vik, C. Nathan Nessle, Festus Njuguna

**Affiliations:** ^1^ Department of Child Health and Pediatrics, Moi University, Eldoret, Kenya; ^2^ Department Pediatrics, Division Hematology-Oncology, University of North Carolina at Chapel Hill, Chapel Hill, NC, United States; ^3^ Academic Model for Providing Access to Healthcare, Eldoret, Kenya; ^4^ Department of Global Health, University of Washington, Seattle, WA, United States; ^5^ Emma Children’s Hospital of the Amsterdam University Medical Center (UMC), Vrije Universiteit, Amsterdam, Netherlands; ^6^ Moi Teaching and Referral Hospital, Eldoret, Kenya; ^7^ Department of Learning Health Sciences, University of Michigan Medical School, Ann Arbor, MI, United States; ^8^ Department of Pediatrics, Division of Hematology-Oncology, Riley Hospital for Children, Indiana University School of Medicine, Indianapolis, IN, United States; ^9^ Department of Pediatrics, Division of Hematology-Oncology, University of Michigan, Ann Arbor, MI, United States; ^10^ Fogarty International Center, National Institute of Health, Bethesda, MD, United States

**Keywords:** supportive care, febrile neutropenia, pediatric oncology, Africa, implementation science, antibiotic

## Abstract

**Introduction:**

Compared to febrile neutropenia (FN), non-neutropenic fever (NNF) episodes in children with cancer have not been associated with severe outcomes. Risk factors for severe outcomes in FN and NNF episodes in children with cancer from low-middle-income countries (LMIC) are incompletely described.

**Methods:**

A prospective, observational cohort study was conducted at a tertiary public referral hospital in western Kenya. Inclusion criteria were age ≤14 years, cancer diagnosis, hospitalized, fever >38.5°C or persistently >38°C. Neutropenia was an absolute count (ANC) <500 K/µL. Severe outcomes were BSI or death. Statistical analysis detected significance between groups and a univariate analysis was conducted.

**Results:**

Of the 99 fevers, 54.5% were NNF episodes. Over 66% of NNF episodes were in patients with solid tumors. More severe outcomes were observed in NNF episodes compared to FN [BSI: 7.4% (4/54) vs. 4.4% (2/45); death: 7.4% (4/54) vs. 4.4% (2/45)], yet no deaths occurred in episodes with BSI. Acute leukemia not in remission (OR= 8.67, 95% CI [CI: 2.3-32.62]; *p*= 0.002) and concern for disease relapse (OR= 14.17, 95% CI [2.08-96.3]; *p*= 0.012) were significantly associated severe outcomes. Time to antibiotic administration (9 hours) did not differ by ANC. Under half (45.5%) of fever episodes had a blood culture ordered, with 93.9% obtained after administration of antibiotics.

**Discussion:**

Non-neutropenic fever episodes had more severe outcomes. Prompt fever management is recommended in all children with cancer treated in an LMIC setting. Pediatric oncology treatment centers in LMICs should rigorously evaluate their fever management clinical practice. Clinical risk factors were identified, but a risk-stratified approach in an LMIC setting is not recommended. Urgent attention is needed to identify areas of clinical improvement.

## Introduction

In high-income countries (HIC), febrile neutropenia (FN) episodes have a bloodstream infection (BSI) incidence of 11-24%, with intensive care unit admissions of 0.9-11% (1–3). Fevers in non-neutropenic (NNF) patients have a lower BSI incidence of 4.4% (4–6). Through advances in supportive care and efforts to improve early detection and management of fever episodes (7, 8), the current estimated mortality from FN episodes is 47 per 1,000, a marked improvement from above 85% in patients with acute leukemia and profound neutropenia at its initial description in the 1960s (9–11). This improvement in fever outcomes facilitates risk stratification of FN (12) and NNF episodes (13) in HICs (6, 14).

In contrast, children with cancer in lower- and middle-income countries (LMICs) are 20–30 times more likely to die from an infection (15–17), which significantly contributes to poor overall survival (20-30%) compared to >85% in HICs (18, 19). While multifactorial in nature, infection-related mortality increases with moderate (>3 hours) and severe (>24 hours) delays in fever management (15, 20, 21). Consequently, reduced-intensity chemotherapy regimens are utilized to mitigate the high risks of infection-related mortality (22). Improved timeliness and management of severe illness in pediatric oncology patients, which included fever episodes, improved outcomes in LMIC (23).

Febrile episodes of pediatric cancer patients have been incompletely described in sub-Saharan Africa (SSA). Regional and country-specific descriptions highlight variability in BSI incidence (14.1% to 29.4%), predominant bacterial isolates, and antibiotic-resistant patterns (16, 24–28). Despite the known association with high mortality, few febrile cancer patients receive timely antibiotic treatment and diagnostic evaluations (16, 25, 26). Tertiary treatment centers in SSA have documented delayed fever management in children with cancer; <16% of febrile patients get blood cultures, and <23% of antibiotics are given within 3 hours of fever detection (15, 26). While risk tools for FN (1, 2, 29, 30) and NNF episodes (6) are validated in HICs, risk factors associated with severe outcomes in FN and NNF episodes have been incompletely described in SSA.

We conducted a mixed methods implementation science study to comprehensively describe the management of FN and NNF episodes to identify areas to improve clinical care (31). Here, we focus on the description of fever episodes, clinical management, and risk factors associated with severe outcomes to highlight the increased incidence of death and BSI in NNF episodes compared to FN episodes in our tertiary, public referral pediatric cancer treatment center in western Kenya.

## Methods

### Study design and definitions

A prospective, convergent mixed methods implementation science study was conducted to describe barriers to and facilitators of fever management in children with cancer at our facility (31). A fever was defined as a temperature of 38°C in 2 consecutive readings at least 1 hour apart or a single fever >38.5°C (32). A non-contact infrared thermometer (Guangdong Genial Technology Co. Limited, Infrared Forehead Thermometer model: T81) was used per routine clinical practice. Neutropenia was an absolute neutrophil count <0.5 K/µL, or 1 K/µL, with anticipation that it would decrease (32). Severe outcomes were BSI (33) or death during the fever episode. A validated FN risk stratification tool by Alexander, et al. (29) recommended by the Children’s Oncology Group was used (12). A high-risk primary oncological disease was acute leukemia in induction, acute leukemia not in remission, infantile leukemia, acute myelogenous leukemia, or non-Hodgkin lymphoma (12, 29).

### Setting

Shoe4Africa at Moi Teaching and Referral Hospital (MTRH) is the largest public children’s hospital in East Africa, located in western Kenya, and serves a population of over 25 million (34). The pediatric oncology program collaborates with international partners to comprehensively improve cancer care (35) through multiple programmatic outreach activities (36), managing over 250 new cases of pediatric cancer annually. The pediatric oncology fever approach at MTRH was adapted from society recommendations: an infectious disease diagnostic evaluation followed by the administration of empiric third generation cephalosporin in NNF episodes or fourth generation cephalosporin in FN episodes (30). Typically, children received only *Pneumocystis jiroveci pneumonia* prophylaxis; routine antibacterial prophylaxis was not utilized. The children with cancer at MTRH receive peripheral administration of chemotherapy without the use of central venous catheters.

### Study population

The study period was from January 2023 to June 2023. To meet inclusion criteria, participants were children with cancer under 14 years of age admitted to MTRH and diagnosed with a new fever episode. Participants were excluded if the first fever was detected as an outpatient, the patient was transferred from an outside facility for fever management, or a fever episode was documented within 7 days of another fever. Written consent was obtained.

### Data collection

Clinical course variables were obtained by manual abstraction of the medical chart. During study planning, clinical variables were identified in several locations: electronic medical records, internal microbiology lab specimen registry, internal pharmacy computer documentation, and over 5 hard copy medical records in the inpatient ward. Therefore, a standardized data collection plan was generated, monitored, and evaluated routinely. Data was collected and stored in a REDCap database for the project (37).

### Statistical analysis

Statistical analyses were conducted using RStudio 2023.06.0 software, with significance defined as a p-value of less than 0.05. Descriptive statistics characterized demographic data. Continuous variables were summarized using medians and ranges to capture the central tendency and variability, while categorical variables were presented as proportions. Univariate analyses identified factors associated with survival and severe outcomes. Variables with significant associations in univariate analysis were considered for inclusion in further multivariate models to adjust for potential confounders. Missing data were handled through appropriate imputation or exclusion based on the extent of missingness and the nature of the analysis.

### Ethics and dissemination

Institutional review board approval was obtained from MTRH (0004273) and the University of Michigan (HUM0225674), and the study was registered with National Commission for Science Technology and Innovation (P/23/22885). Results were shared with local and national policy leadership, local stakeholders, and presented at international academic conferences. Data is available upon request.

## Results

### Fever episode demographics and characteristics

The characteristics of the 99 fever episodes are described in **Tables 1**, **2**. Of the 99 fever episodes, 54% were NNF, and 53.5% were in male children, with an average age of 6 years. The primary oncologic diagnosis was significantly different between the two groups (*p*= <0.001) as NNF episodes were more likely to be diagnosed with non-CNS solid tumors (66.7%; 36/54), while a greater proportion of FN episodes were acute leukemia (51.1%; 23/45) compared to NNF (14.8%; 8/54). Acute leukemia in induction had more FN (80%; 12/15) while a concern for disease relapse was described in 2 FN episodes and 3 NNF episodes.

**Table 1:** Fever episode demographics.

Variable	Total	Febrile Neutropenia	Non-Neutropenic Fever	*p*-value
Total number of fever episodes	99	45 (45.5)	54 (54.5)	
Age (mean in years)		6.1	5.9	
Gender				
Male	53 (53.5)	22 (48.9)	31 (57.4)	0.260
Female	46 (46.5)	23 (51.1)	23 (42.6)	
Primary Oncology disease				
ALL	22 (22.2)	17 (37.8)	5 (9.3)	<0.001
AML	9 (9.1)	6 (13.3)	3 (5.6)	
Hodgkin Lymphoma	1 (1)	0 (0)	1 (1.9)	
Non-CNS solid Tumor	52 (52.5)	16 (35.6)	36 (66.7)	
Non-Hodgkin Lymphoma	12 (12.1)	6 (13.3)	6 (11.1)	
Primary brain tumor	3 (3)	0 (0)	3 (5.6)	

ALL, acute lymphoblastic leukemia; AML, acute myelogenous leukemia; non-CNS, non-central nervous system.

**Table 2:** Infectious cause and clinical outcomes of fever neutropenia and non-neutropenic fever episodes.

Variable	Total group	Febrile Neutropenia	Non-Neutropenic Fever	*p*-value
Number of episodes	99	45 (45.5)	54 (54.5)	
Infectious cause of fever				
Not identified	79 (79.8)	41 (41.4)	38 (38.1)	0.409
Blood stream infection	6 (6.1)	2 (2)	4 (4)	
Malaria parasite infection	5 (5.1)	1 (1)	4 (4)	
Skin/soft tissue infection	3 (3)	0	3 (3)	
Urinary tract infection	1 (1)	0	1 (1)	
Invasive fungal infection	2 (2)	0	2 (2)	
Tuberculosis	0	0	0	
Other	3 (3)	1 (1)	2 (2)	
Severe comorbidities				
Yes (High Risk)	61 (61.6)	32 (71.1)	29 (53.7)	0.058
No (Low Risk)	38 (38.4)	13 (28.9)	25 (46.3)	
Historic risk Factors	50 (50.5)	27 (60)	23 (42.3)	
Outcome of fever episode				
BSI, Recovery	6 (6.1)	2 (4.44)	4 (7.4)	0.280
Death	6 (6.1)	2 (4.44)	4 (7.4)	
Recovery from fever episode	93 (93.9)	43 (95.6)	50 (92.3)	

Severe comorbidities were clinical criteria present at the time of fever detection such as respiratory distress, low oxygen, chills, shock, hypotension, altered mental status, kidney injury, liver injury, vomiting, severe abdominal pain, severe pain from mucositis, or a concern for a focal infection requiring parenteral antibiotics. Historical risk factors were down syndrome, age <1 year, induction for leukemia, leukemia not in remission, concern for cancer relapse or progression, diagnosis of acute myelogenous leukemia, infantile leukemia, or non-Hodgkin Lymphoma. BSI, blood stream infection.

### Infectious outcomes and deaths in fever episodes

There were no significant differences in microbiologically defined infections between FN and NNF groups (*p*=0.409). An infectious cause was not identified in 79.8% of cases. Malaria was diagnosed in 5.1% of fever episodes and all blood cultures from BSI episodes were collected within 24 hours of fever detection. Non-neutropenic fever episodes had twice as many BSI [7.4% (4/54) vs. 4.4% (2/45)] and deaths [7.4% (4/54) vs. 4.4% (2/45)] compared to FN episodes (*p*=0.280). All patients with an episode of a BSI recovered. The 4 NNF episodes which resulted in death occurred in children diagnosed with acute myelogenous leukemia not in remission, 2 episodes of nephroblastoma, and Burkitt lymphoma are described in **Supplementary Material 1**.

### Risk factors for a blood stream infection or death

Severe comorbidities (e.g., hypoxia, shock, altered mental status, etc) were analyzed separately from historic risk factors (e.g., leukemia in induction, acute myelogenous leukemia, etc). In NNF episodes (n=54), 53.7% had a clinical severe comorbidity at presentation, and 42.3% had a historically high-risk oncologic diagnosis. (**Table 2**) Univariate analysis of factors contributing to either death or BSI is summarized in **Table 3**. Acute leukemia not in remission (OR= 8.67, 95% CI [CI: 2.3-32.62]; *p*= 0.002) and concern for disease relapse (OR= 14.17, 95% CI [2.08-96.3]; *p*= 0.012) were significantly associated with increased risk of a severe outcome. When stratified by ANC, induction for acute leukemia and concern for disease relapse has an increased risk for a severe outcome in FN episodes, while a high-risk primary disease and acute leukemia not in remission were significantly worse in NNF episodes. No fever episode was managed in the intensive care unit.

**Table 3:** OR of clinical risk factors for severe outcomes in febrile episodes in children with cancer.

Characteristic	Total Group		Febrile Neutropenia		Non-Neutropenic Fever	
	Odd Ratio (95% CI)	*P-*value	Odd Ratio (95% CI)	*P-*value	Odd Ratio (95% CI)	*P-*value
Age	2.25 (0.41-12.29)	0.524	4.26 (0.19-98.1)	0.185	1.33 (0.2-8.71)	0.440
Severe Neutropenia	0.56 (0.16-2)	0.538	NA		NA	
High Risk Primary Disease	1.03 (0.16-6.62)	0.350	0.67 (0.049-9.022)	0.890	3 (0.15-59.89)	0.052
Induction for acute leukemia	3.45 (0.89-13.41)	0.082	10.67 (0.99-115.36)	0.052	3.14 (0.25-39.44)	0.388
Acute Leukemia not in remission	8.67 (2.3-32.62)	0.002	12.38 (1.13-135.24)	0.039	27 (2.34-311.19)	0.008
Concern for disease relapse	14.17 (2.08-96.3)	0.012	83 (3.080-223.416)	0.001	3.14 (0.25-39.44)	0.388
Severe comorbidities	2.02 (0.51-7.99)	0.362	1.24 (0.12-13.16)	0.998	3 (0.55-16.45)	0.262

Severe comorbidities were clinical criteria present at the time of fever detection such as respiratory distress, low oxygen, chills, shock, hypotension, altered mental status, kidney injury, liver injury, vomiting, severe abdominal pain, severe pain from mucositis, or a concern for a focal infection requiring parenteral antibiotics. Severe neutropenia was an absolute neutrophil count <0.5 K/µL, or 1 K/µL, with anticipation that it would decrease. High risk primary disease was acute myelogenous leukemia, infantile leukemia, or non-Hodgkin Lymphoma.

### Timeliness of fever management by neutrophil count

**Figure 1:**
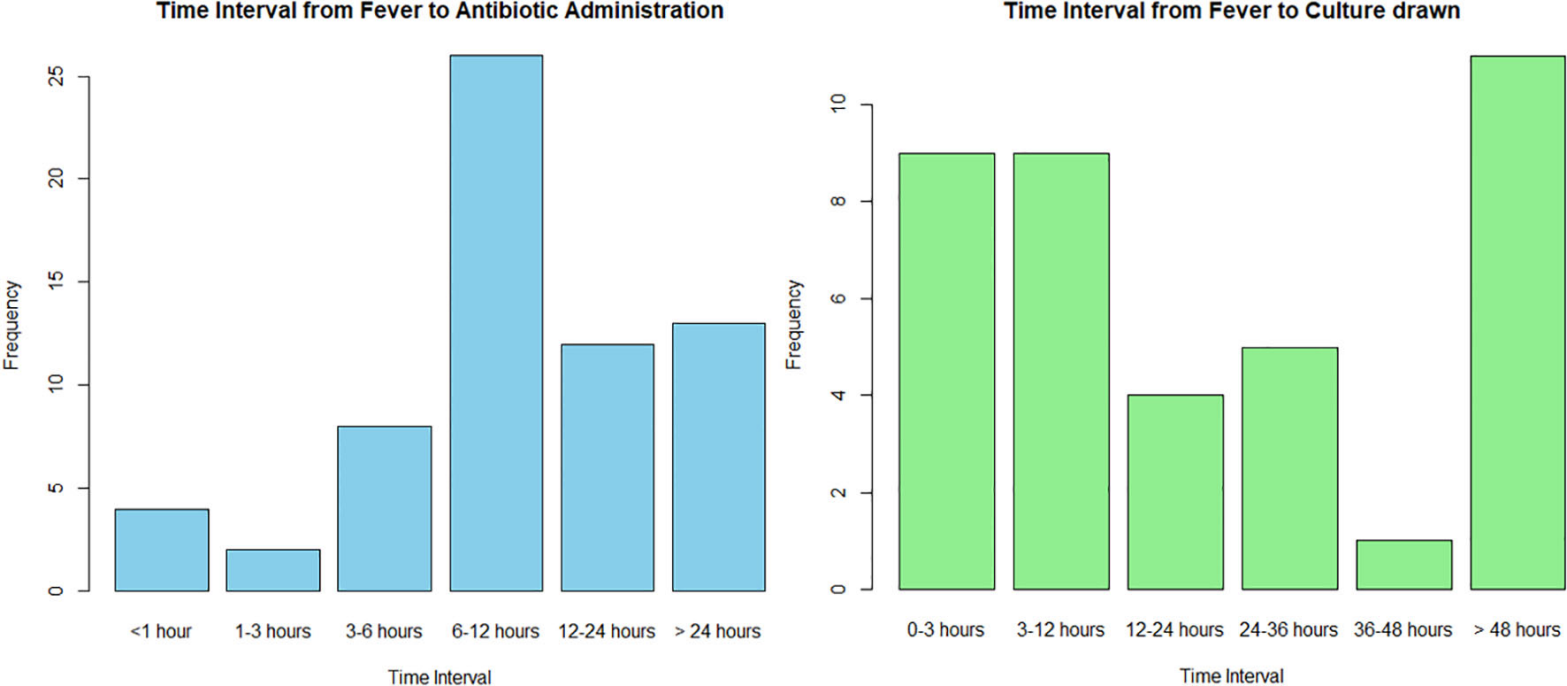
These histograms describe the elapsed time from fever detection to antibiotic administration (blue) and from fever detection to a blood culture drawn (green) for the fever episode.

Antibiotics were administered in 88.9% of fever episodes. The median time from fever detection to antibiotic administration (TTA) was 9 hours without difference by ANC. (**Table 4**) Only 7.1% of antibiotics were administered within 3 hours of fever detection and 20.3% were administered 24 hours after fever detection (**Figure 1**). Blood cultures were drawn in 45.5% of episodes with 93.9% obtained after administration of antibiotics; no repeat cultures were observed. The median time from fever detection to blood culture draw was 15 hours, but longer in FN (16.8 hours vs. 8.3 hours). Over 37.8% of blood cultures drawn occurred 24 hours after fever detection with 64.7% (11/17) drawn after 48 hours.

**Table 4:** Blood culture and antibiotic management workflows of inpatient fever episodes.

Variable	Total	Febrile Neutropenia	Non-Neutropenic Fever
Number of fever episodes	99	45 (45.5%)	54 (54.5%)
Blood culture workflow			
Median time to blood culture draw	15 hrs [0.06-271.2 hrs]	16.8 hrs	8.3 hrs
Blood cultures drawn	45 (45.5%)	25 (57.8%)	20 (40.7%)
Blood culture before antibiotic administration	8 (6.1%)	3 (6.7%)	5 (10.8%)
Antibiotic workflow			
Median Time to antibiotic administration [IQR]	9 hrs [8-16.5]	8.6 hrs [7.9-17.1]	10.3 hrs [8-16.5]
Antibiotics administered	88 (88.9%)	43 (95.6%)	45 (83.3%)
Antibiotics administered within 3 hours of fever	7 (7.1%)	2 (4.4%)	5 (9.3%)

## Discussion

This observational study in hospitalized children with cancer in Kenya improves the description of the inpatient management of pediatric cancer fever episodes in several ways. First, this study describes an increased incidence of death and BSI in NNF episodes compared to FN episodes in children with cancer, providing, to our knowledge, the first description of severe outcomes in NNF episodes in an SSA cohort. This study also evaluated a Children’s Oncology Group FN risk stratification tool (12, 29) to describe statistically significant clinical factors with increased odds for a severe outcome in both groups. We also provided a detailed description of fever management for both FN and NNF cohorts at our large, tertiary public referral center, which revealed a discrepancy in our internal protocol and actual clinical practice. Overall, this study serves to highlight areas for future research and draw urgent attention to improving the management of FN and NNF episodes in children with cancer in SSA.

The BSI incidence in FN episodes of our study is lower than in existing studies in SSA (14-29.4%) (13). Our study’s objective was to describe the current clinical practice; no fever episode had sequential blood cultures drawn. Therefore, commensal bacterial isolates (e.g., coagulase negative Staphylococcus species) were not documented as BSI events and, possibly related to the absent use of central venous catheters, our study suggests the BSI incidence of commensal isolates as the microbiologically defined infectious cause of fever in a child with cancer might be lower than other reports from SSA (15, 26, 38). However, the BSI incidence (6%) in our cohort is confounded by the large number of blood cultures drawn after broad-spectrum antibiotic administration (93.9%) and missing culture data (45.5% with a blood culture drawn), yet these workflow observations are similar to the literature from the Philippines (39) and sites in SSA (15, 40). Therefore, blood culture results are difficult to interpret in children with cancer treated in LMIC settings that administer chemotherapy without the use of central venous catheters facing similar management workflow challenges.

Our description of inpatient treatment delays in TTA and the collection of a blood culture from the time of fever detection aligns with literature. In our cohort, the median TTA was 9 hours with only 7.1% receiving antibiotics within 3 hours. A study of adults with cancer in Uganda reported a median TTA of 3 days (40), while a pediatric oncology FN report in SSA observed <23% of antibiotics administered within 3 hours of fever detection (15). Many fever episodes have antibiotics administered 24 hours after fever detection, emphasizing the association of increased infection-related mortality to delays in TTA (15, 17, 40). Some studies have shown efforts to decrease TTA have improved fever outcomes (8). A pediatric FN study in the US concluded that a TTA <1 hour improved outcomes in those with severe illness or comorbidities at presentation (41), but it remains to be seen if decreasing the TTA without improving other supportive care measures (e.g., sepsis screening, shock management, access to intensive care unit) is solely sufficient to decrease fever deaths in children with cancer in a low-resourced treatment setting. Blood cultures were drawn in 45.5% of episodes, with most obtained after antibiotics in our cohort. The proportion of fever episodes with a culture drawn was higher compared to pediatric FN reports from Ethiopia (9.6%) (16) and a multicenter SSA study (15%) (15), but lower than a report from the Philippines (87%) (39). To our knowledge, this study is the first to report TTA and blood culture workflows for pediatric NNF episodes in an SSA cohort. Most published literature from SSA described FN episodes, and dedicated descriptions of NNF episodes are lacking.

Our study found a higher incidence of severe outcomes (death, BSI) in NNF episodes compared to FN. In contrast to existing literature from the US, our BSI incidence (7.4% vs 4.1%) and mortality rate (7.4% vs 0.2%) were higher (6). We postulate that it is possible the clinical practice at our center was influenced by existing NNF literature from HICs to produce a bias that NNF episodes have good outcomes that, when combined with supportive care gaps in fever management, contributed to these surprising results. Additionally, a BSI risk calculator in NNF episodes has been validated in the US (4, 6), yet the feasibility and performance of the model in an LMIC setting are unknown. For example, often, the ANC is not available at fever detection and may take over 24 hours to result, and children are unlikely to have a central venous catheter in place, both of which are required variables to complete the risk calculation (6). For FN episodes, most risk stratification evidence is validated in HICs (12, 14). A study in India evaluated FN risk factors among children with acute lymphoblastic leukemia (42), yet the model poorly performed during validation (43). ​We applied the clinical risk factors from a Children’s Oncology Group recommended tool (12, 29) and found several clinical factors (e.g., leukemia in induction, leukemia not in remission, concern for relapse) with increased odds for a severe outcome in both FN and NNF cohorts. However, other aspects of care that support fever management, such as effective sepsis screening (e.g., a pediatric early warning system), favorable nurse-to-patient ratios, and access to a pediatric intensive care unit, are not accessible in our large, tertiary public referral hospital and may also be lacking in similar LMIC settings. In contrast, pediatric oncology treatment centers in HICs -where the majority of FN and NNF risk stratification literature is validated- are likely to have established care capacity in these areas, which may confound the interpretation and limit the application of existing risk stratification literature to an LMIC setting.

This study has several strengths. The external validity of our study is corroborated when compared to existing literature. We provide novel insights into the risk factors of FN and NNF episodes and describe the increased incidence of severe outcomes in the NNF cohort, expanding the literature in these areas in children with cancer in an LMIC setting. While the sample size was not powered to detect statistical significance, the analysis did show risk factors with statistical significance and severe outcomes that trended toward significance. The study design provides valuable insight into the actual fever management in practice, exposing pragmatic gaps to improve management. This study was conducted at a large, tertiary public referral center in Kenya which may allow the generalizability of our results to other tertiary public referral centers in SSA as the resources available may be similar.

However, this study is not without limitations. The study was designed to describe the clinical practice of fever episodes rather than determine differences in clinical outcomes between FN and NNF subgroups. However, even though our study was not powered for statistical significance which may limit the comparison of the subgroups, the results emphasize the urgent need to improve the description of NNF episodes in SSA. Secondly, the BSI incidence reported is lower than existing literature, yet the management patterns (e.g., low proportion of blood cultures drawn, blood culture drawn after antibiotic administration) described in our study, although similar to past reports (15, 39), confound the conclusions for BSI events. Additionally, while we currently show several clinical factors with increased odds of a severe outcomes, there are confounding factors mentioned above (e.g., nursing ratio, sepsis management), and it is not known how these factors impact the clinical risk factors. Therefore, it is possible that the clinical risk factors for FN and NNF episodes may change with efforts that increase clinical care capacity (e.g., improving TTA, implementing a sepsis screen), making risk stratification challenging in LMIC settings. There is a need to improve the description to explain the delays of antibiotic and blood culture workflows for children managed in low-resourced treatment settings, which may facilitate the identification of strategies to improve management (44).

This study highlights the urgent need to improve the management of FN and NNF episodes in children with cancer in SSA. While severe outcomes of FN are well described in LMICs, our study suggests NNF episodes have a higher incidence of severe outcomes. Therefore, we encourage clinicians in LMICs to give prompt fever management to any child with cancer, recognizing severe outcomes are found in both FN and NNF episodes. Secondly, risk stratification literature generated from HIC for FN or NNF episodes should be interpreted with caution in an LMIC setting due to the difference of other supportive care fever management measures. Also, risk factors may change with the implementation of strategies to improve fever management (e.g., implementation of a sepsis screen, decreasing TTA, antibiotic selection informed by local antibiogram, etc.). Pediatric oncology treatment centers in an LMIC setting should strongly consider rigorously evaluating their fever management clinical practice to identify impactful, locally tailored interventions to improve fever management. Finally, future studies should explore the practice habits to improve understanding of treatment delays.

## Conclusion

Non-neutropenic fever episodes have a higher incidence of severe outcomes compared to FN episodes in children with cancer in a tertiary public referral hospital in Kenya. Prompt management of fever episodes in all children with cancer in SSA is recommended, and there is a need to improve the description of NNF episodes in LMICs. While clinical risk factors are associated with severe outcomes in both groups, the clinical implications are challenging in an LMIC as the risk factors are likely to change with efforts to increase care capacity. A risk-stratified approach for FN or NNF episodes in low-resourced treatment centers in an LMIC is not recommended. Studies are needed to improve the understanding of barriers and facilitators to timely fever management in children with cancer in an LMIC setting.

## Data Availability

The raw, de-identified data supporting the conclusions of this article will be made available by the authors, contingent on the approval of the institutional ethical review boards, ensuring that all shared resources adhere to ethical standards and respect participant confidentiality.
