# Supplementary material for: Severe outcomes and risk factors of non-neutropenic fever episodes in hospitalized children with cancer in Kenya
Source: Front Oncol. 2025 May 12;15:1575714. doi: 10.3389/fonc.2025.1575714 (PMC12104272; doi:10.3389/fonc.2025.1575714)
Supplement: Supplementary file 1 [file Table1.pdf]

Table 4: Characteristics of fever episodes with death as outcome						
**Variable**	Death #1	Death #2	Death #3	Death #4	Death #5	Death #6
**Age**	16 months	13 years	7 years	3 years	3 years	4 years
**Gender**	Female	Female	Female	Male	Male	Female
**Primary Oncology disease**	Nephroblastoma	Biphenotypic (B/Myeloid) Acute Leukemia	Burkitt Lymphoma	Nephroblastoma	ALL	AML
**Neutropenia at time of fever**	No	Yes	No	No	Yes	No
**Neutropenia at time of death**	Yes	Yes	Unknown	Yes	Yes	No
**Date of death after fever onset**	3 days	9 days	3 days	7 days	4 days	23 days
**Risk Factors**						
**Leukemia in induction**	N/A	Yes	No	N/A	Yes	No
**Leukemia not in remission**	N/A	Yes	N/A	N/A	No	Yes
**Concern for relapse or progression**	No	Yes	Yes	No	No	No
**Severe comorbidities at time of fever**	2	2	3	2	2	0
**Severe comorbidities at time of death**	4	4	Unknown	4	2	3
**Blood stream infection**	No	No	No	No	No	No
**Infectious related cause of death**	Yes	Yes	Unknown	Possible	Yes	Possible
